# Results of total knee arthroplasty for painless, stiff knees

**DOI:** 10.1186/s43019-020-00081-0

**Published:** 2020-11-17

**Authors:** Young-Joon Choi, Dong-Kyo Seo, Ki Won Lee, Ho Jong Ra, Hyun Wook Kang, Jin Kyung Kim

**Affiliations:** Department of Orthopaedic Surgery, GangNeung Asan Hospital, University of Ulsan, College of Medicine, 38 Bangdong-gil, Sacheon-myeon, Gangneung, Gangwon-do 25440 South Korea

**Keywords:** Knee, Stiff, Ankylosis, Total knee arthroplasty

## Abstract

**Background:**

Stiff knees, like completely ankylosed or arthrodesed knees, can be painless. Total knee arthroplasty (TKA) for these painless, stiff knees is technically demanding. However, it can correct the alignment and advance the range of motion to improve quality of life. So, we reviewed the preoperative and postoperative results of functional and pain scores, range of motion (ROM) and complications in painless, stiff knees treated by TKA.

**Methods:**

Fifteen painless, stiff knees underwent TKA from January 1998 to January 2017. The mean follow-up period was 15.4 (2.4–22.2) years. All the knees were completely ankylosed or arthrodesed. Clinical outcome and complications were evaluated using medical record review, serial plan radiography, ROM assessment, Knee Society score (KSS), Knee Society function score (FS), and a visual analog scale for pain (VAS).

**Results:**

All patients were satisfied with their operated knees. Mean KSS and FS scores were improved from 36 and 50.9 to 76.9 and 67.2, respectively (*P* < 0.001 and *P* = 0.01). The mean ROM increased from 0º preoperatively to 77.6º (15–130) at the final follow-up (*P* < 0.001). The mean VAS had worsened from 0 preoperatively to 0.2 postoperatively, however it was not significant (*P* = 0.1). Major postoperative complications were reported in five of the knees (33.3%).

**Conclusions:**

The results of TKA for painless, stiff knees were satisfactory with improved ROM and quality of life. Although some patients had mild pain and complications postoperatively, they were satisfied with the result. However, our study recommends that surgeons should consider the high rate of complications in the completely ankylosed or arthrodesed knees.

**Level of evidence:**

A retrospective case series, Level IV.

## Introduction

A knee can be stiffened from various etiologies. Common etiologies are post-traumatic arthritis, pyogenic arthritis, rapidly progressing arthritis, rheumatoid arthritis, ankylosing spondylitis, and psoriatic arthritis, and untreated tuberculosis can be a reason in specific global regions [1–3].

Knee range of motion (ROM) is important for the activities of daily living. When the knee is stiff and in an improper position, walking disturbance, limping, and severe discomfort in daily life may be induced. Most patients have discomfort in sitting and a psychologic burden of disability. At least 60–70º of flexion is needed for a normal gait, 90º for stair-climbing, and 105º for rising from a low chair. So, total knee arthroplasty (TKA) can be a choice for patients expecting an improved quality of life by reducing the pain and increasing the ROM of the knee [1–6].

However, stiff knees, like completely ankylosed or arthrodesed knees, are sometimes painless. Surgery for the painless, stiff knee can be controversial because it improves the ROM only. There are a few published reports regarding TKA in ankylosed or arthrodesed knees [3, 7–9]. However, there are few published reports about TKA for painless and completely ankylosed or arthrodesed knees. Our hypothesis was that TKA for painless, stiff knees could be an effective treatment method to improve the patient’s quality of life, even though it does not correlate with pain reduction. In this study, we reviewed the preoperative and postoperative results of functional and pain scores, ROM, and complications in painless, stiff knees treated by TKA.

## Materials and methods

### Patients

Our Institutional Review Board approved this study. The inclusion criterion was painless, stiff knees without any ROM. Completely ankylosed, spontaneously bony fused, or iatrogenically arthrodesed knees were included. Complete ankylosis was defined as spontaneously ankylosed knees with no ROM even though a gap was seen on radiographic images. The exclusion criteria were stiff knees with a ROM like partial ankylosis, pseudo-arthrodesed knees, knees of patients with systemic musculoskeletal disease, brain damage, and spinal disorder, which discourages improved ROM after TKA, were excluded. Knees with active infection at the time of surgery were also excluded.

Fifteen knees (15 patients) that underwent TKA from January 1998 to January 2017 were evaluated. The mean follow-up period was 15.4 (2.4–22.2) years. The detailed preoperative demographic data is described in Table 1 and the etiology of the diseased knees in Table 2. The mean preoperative ROM was 0.0 ± 0.0º, and the mean fused angle was 11.6 ± 17.9º (range; 0–50º)in a flexion position. Ten knees (66.6%) had previous operative history. Of these, seven knees (46.6%) were fused by knee arthrodesis. The mean duration of experiencing a stiff knee before operation was 17.5 ± 12.1 (2–45) years. No patient complained of any knee pain. Five knees showed marked quadriceps atrophy at operation (33.3%) and one knee had undergone patellar excision at a previous operation.

**Table 1:** Preoperative demographic data of patients

	Mean value	Range
Number of knees (patients)	15 (15)	
Mean follow-up period (years)	15.4 ± 6.1	(2.4–22.2)
Mean duration of stiffness (years)	17.5 ± 12.1	(2–45)
Mean age (years)	71.4 ± 12.8	(46–94)
Mean height (m)	1.56 ± 0.08	(1.4–1.6)
Mean body weight (kg)	59.9 ± 6.5	(48–66.3)
Mean body mass index (kg/m^2^)	24.4 ± 2.9	(21.5–28.9)

The values are given as the mean ± standard deviation, with the range in parentheses

*Kg* kilograms, *M* meters

**Table 2:** Etiology of stiffness and the number of knees studied

Etiology	Number of knees	Percentage
Osteoarthritis	4	26.6%
Sequelae of tuberculosis	4	26.6%
Rheumatoid arthritis	3	20%
Post-traumatic arthritis	3	20%
Sequelae of pyogenic arthritis	1	6.6%
Total number of knees	15	100%

Radiologic evaluation was performed in a standing anteroposterior (AP), supine lateral position according to the Knee Society guidelines preoperatively and at 2 weeks, 3 months, and 1 year postoperatively, and annually thereafter [10]. A skyline patellar view and full-flexion, lateral knee plain radiograph was added postoperatively. The clinical outcome was calculated using the Knee Society Knee score (KSS), the Knee Society Function score (FS), ROM, and a visual analog scale for pain (VAS) preoperatively and at the last follow-up. The ROM, KSS, and FS was measured by a senior author (Y-J. C).

### Operative technique and rehabilitation

All procedures were performed by one senior surgeon under general anesthesia and using pneumatic tourniquet control. A midline incision or a previous skin incision was used together with a medial parapatellar capsulotomy. Quadriceps turndown and tibial tuberosity osteotomy was added to avoid patellar tendon avulsion and facilitate exposure. A detailed number of knees for various exposure methods is described in Table 3. The bony ankylosis was taken down with attention to preserving the bone stock and medial and lateral soft-tissue sleeves. An intramedullary guide was used for the femur and an extramedullary instrumentation for the tibia. The cutting level of the distal femur was decided by reference to the epicondyles, if available, or using spacers after tibial cutting.

**Table 3:** Additional exposure methods for stiff knees

Methods	Number of knees	Percentage
Quadriceps turndown	13	86.6%
Tibial tuberosity osteotomy	2	13.3%
Total number of knees	15	100%

Soft-tissue balancing was performed using a spacer block and the final stability was assessed after the trial component was placed. If there was major instability in the coronal plane or a flexion/extension imbalance, a valgus-varus constrained (VVC) prosthesis was selected. Other criteria for using a VVC prosthesis were marked osteoporosis and a prolonged bony ankylosis with attenuated collateral ligaments. We used a VVC prosthesis in 12 knees (80%). The posterior-stabilized (PS) type was used in other knees without described conditions for VVC type.

**Fig. 1:**
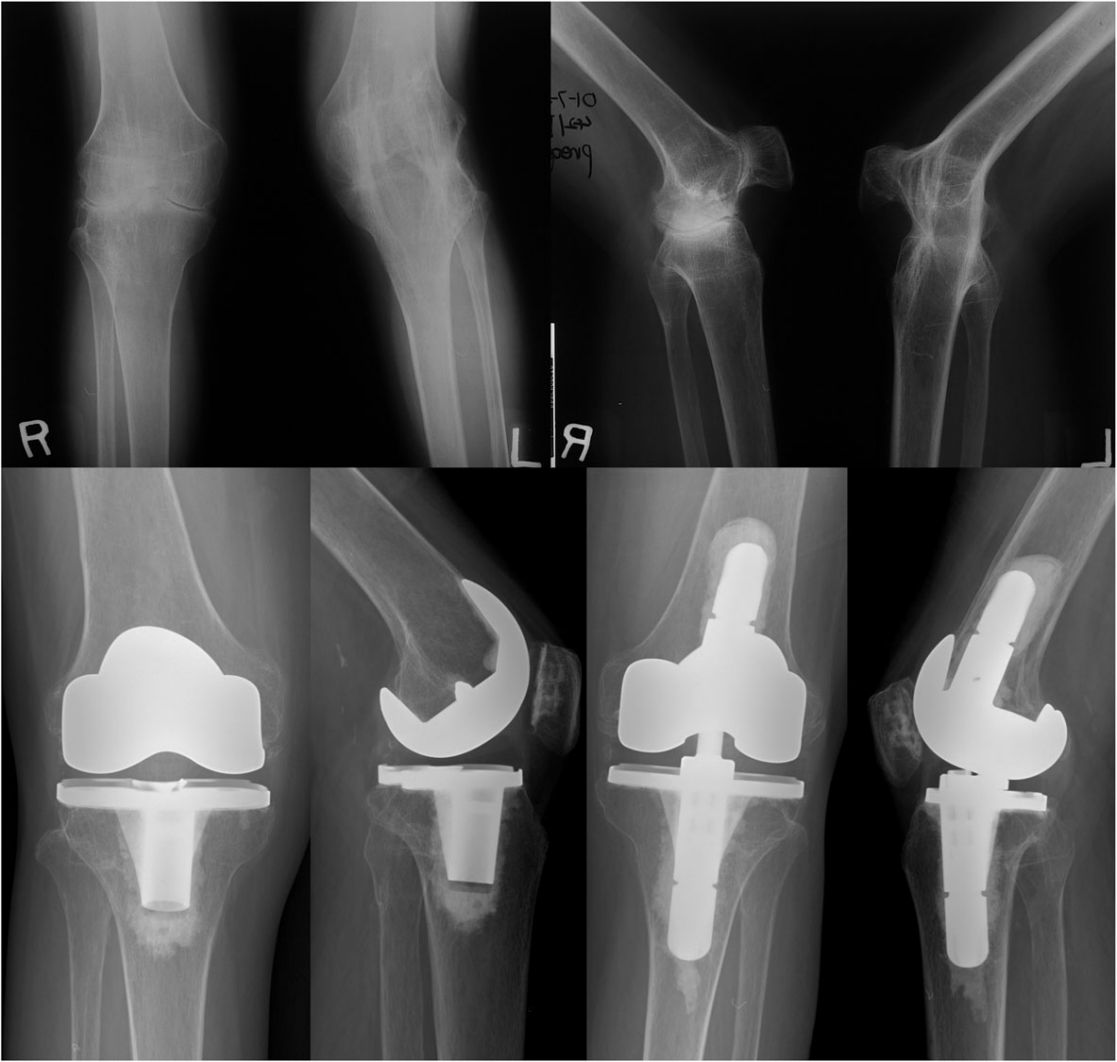
A 44-year-old female patient who had bilateral knee pain due to rheumatoid arthritis. The right knee showed 15º range of motion (ROM) (range; 30–45º) and the left knee was spontaneously ankylosed in 40º of flexion. In the right knee, a PS type (Nexgen LPS) prosthesis was used, and in the left knee, a valgus-varus constrained (VVC) type (Nexgen LCCK) prosthesis was used. The right-knee arc of motion improved from 15 to 85º (range; 0–85º) and the left-knee arc of motion improved from 0 to 85º (range; 0–85º). No complications were noticed during the 16-year follow-up. The left knee (complete ankylosis) was included in this study

After the trial components were inserted, stability was tested. According to the stability, types of implant were selected. No hinged prosthesis was used in this series (Fig. 1). The types of prosthesis are described in Table 4. All components were implanted with cement. The patella was resurfaced in all cases. Capsule closure was performed in a 60° flexion position.

**Table 4:** Types and subtypes of prosthesis

Prosthesis	Number of knees
Valgus-varus constrained (VVC)	12 (80%)
Posterior-stabilized (PS)	3 (20%)
Total number knees	15 (100%)

No splint or brace was used for protection even in the cases using quadriceps turndown for exposure. Continuous passive motion exercise was started from the first day after the operation for every patient. Weight-bearing was started on the second day after surgery with a walker.

### Statistical analysis

The paired T test or Mann-Whitney *U* test was used to compare the preoperative and postoperative changes of continuous variables. The chi-square or Fisher’s exact test was used to compare the categorical variables. A *P* value < 0.05 was considered as statistically significant. Statistical analyses were performed using SPSS, version 21.0 (SPSS Inc., Chicago, IL, USA).

## Results

All patients were satisfied with their operated knees. Mean KSS and FS scores were improved from 36 and 50.9 to 76.9 and 67.2, respectively (*P* < 0.001 and *P*= 0.01). The mean ROM increased from 0 to 77.6 ± 36.4º (15–130º) at the final follow-up (*P* < 0.001). The mean postoperative ROM range was from 6.6 ± 13.4º (0–50º) to 85.0 ± 34.5º (30–130º). At final follow-up, 3 of 15 knees (20%) showed mild pain (VAS 1 or 2). The mean VAS had worsened from 0 to 0.2; however, this was not significant (*P* = 0.1). Detailed mean values of clinical outcome scores are described in Table 5.

**Table 5:** Comparison of preoperative and postoperative clinical outcome

Results	Preoperative	Last follow-up	*P* value
Mean KSS	36.0 ± 21.0 (3–65)	76.9 ± 11.4 (60–100)	*P* < 0.001
Mean FS	50.9 ± 21.0 (5–70)	67.2 ± 19.5 (30–100)	*P*= 0.01
Mean ROM	0 ± 0 (0–50)	77.6 ± 36.4 (15–130)	*P* < 0.001
VAS	0	0.2 ± 0.5 (0–2)	*P*= 0.1

The values are given as the mean ± standard deviation, with the range in parentheses

*FS* Knee Society function score, *KSS* Knee Society score, *ROM* range of motion, *VAS* visual analog scale

**Fig. 2:**
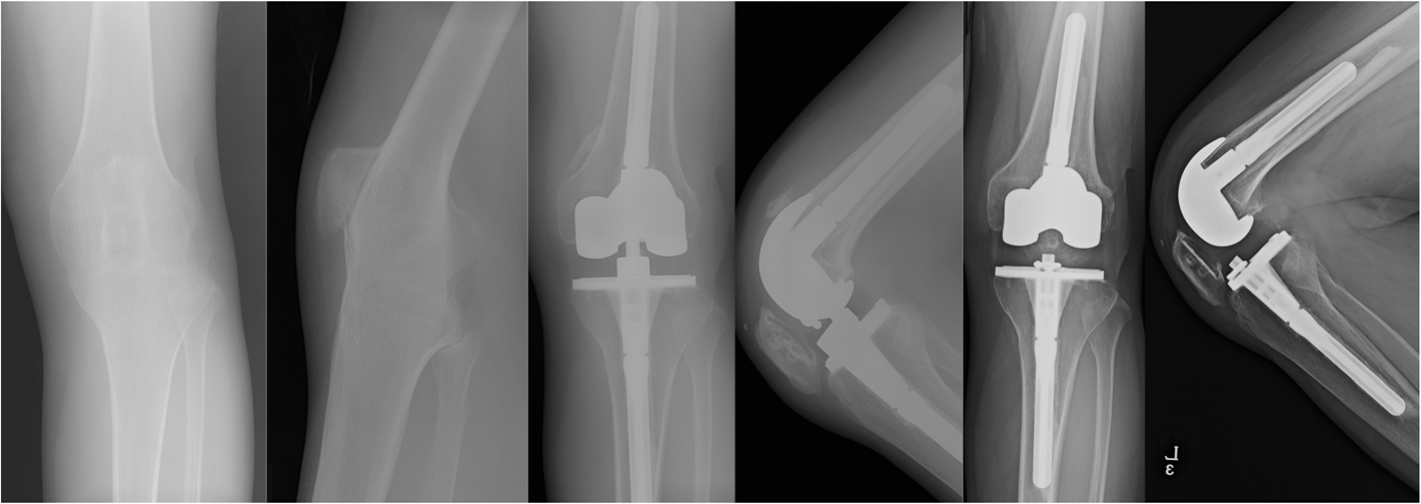
A 31-year-old male patient who had a history of arthrodesis due to tuberculosis infection of the left knee at the age of 17 years. His right knee was ankylosed in 15º of flexion. Take down and total knee arthroplasty (TKA) with a valgus-varus constrained (VVC) prosthesis (Nexgen LCCK) were performed. At 51 months after the key operation, the polyethylene insert was dislocated with breakage of the locking screws after sea-swimming. After exchanging the polyethylene insert with a posterior-stabilized (PS) type, no further complications occurred, with an arc of motion of 0–130º. (Left 2 images, preoperative anteroposterior and lateral views; middle 2 images, postoperative 4 years; right 2 images, postoperative 14 years anteroposterior and lateral views)

**Fig. 3:**
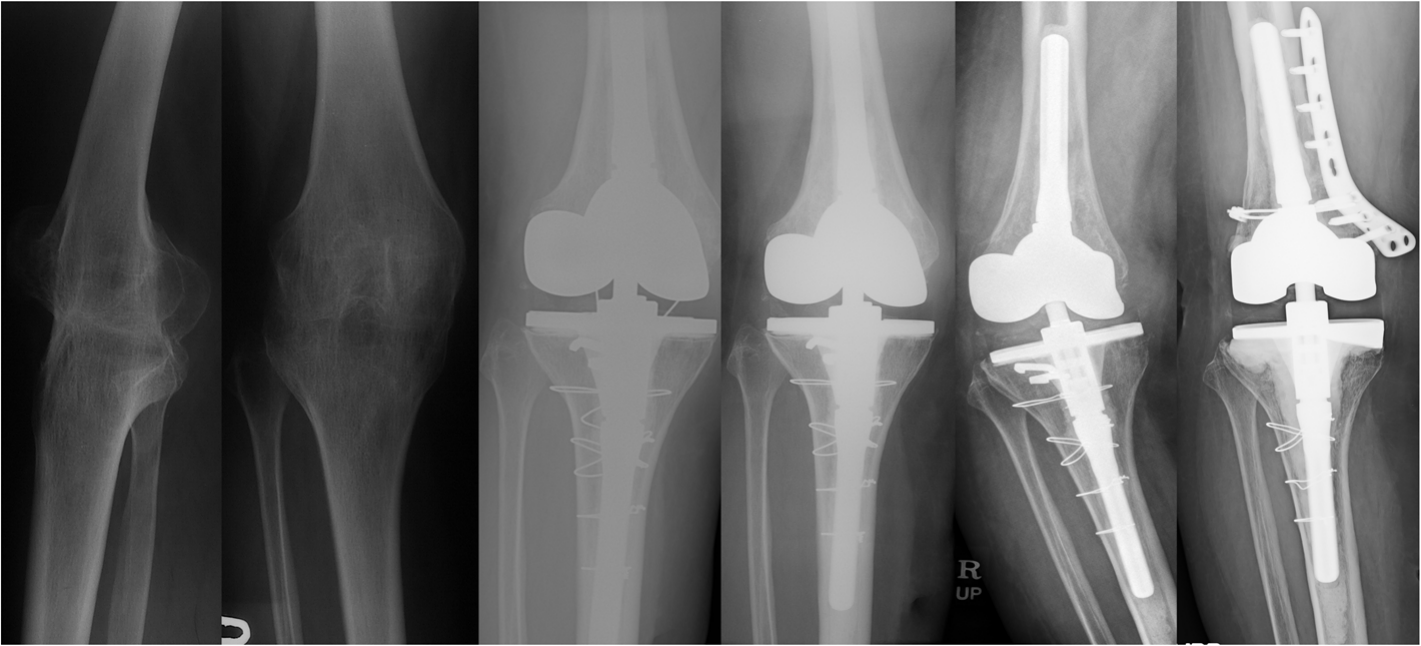
A 60-year-old female patient who had a history of arthrodesis due to severe trauma of the right knee at the age of 50 years, had an ankylosed knee in the 10º flexion position. The right knee was operated on with a valgus-varus constrained (VVC) type (Nexgen LCCK) prosthesis. A tibial tuberosity avulsion fracture occurred in the operation, which was fixed using the tension-band wiring technique. The postoperative arc motion was improved from 0 to 65 (range; 15–80º) with 15º of extension lag. In the 16-year follow-up, polyethylene wear and a fracture of the medial femoral condyle were noticed. A revision was performed with the same type of implant and an internal fixation of the fracture fragment

Major postoperative complications were reported in five knees (33.3%, Table 6). One intraoperative complication occurred. There was one tibial tuberosity avulsion that was fixed using the tension-band wiring technique with nonabsorbable material. In the mid-term period (postoperative 5 years), a dislocation of VVC type polyethylene occurred with breakage of the locking screws in one knee during sea-swimming, and the insert was changed. (Fig. 2) In the late period (postoperative 5 years), there were two cases of a periprosthetic joint infection. Revision was performed with an entire component change via a two-stage operation using a cement spacer. A mild extension lag (10 and 15º) remained in these knees. One patellar fracture occurred during physiotherapy which was treated conservatively with a 4-week immobilization, which showed a severe extension lag of 50º. One knee which had an intraoperative tibial tuberosity avulsion, showed no extension lag at follow-up but aseptic loosening with a distal femoral fracture developing at 16 years postoperatively. (Fig. 3). The details of the demographic data, etiology, ROM, prosthesis type, and complications for all knees are described in Table 7.

**Table 6:** Major complications

	Complication	Number of knees
Intraoperative	Tibial tuberosity avulsion	1
Mid-term (POD < 5 years)	Polyethylene-insert dislocation	1
Late (POD > 5 years)	Periprosthetic joint infection	2
	Patellar fracture	1
	Aseptic loosening (same knee with intraoperative tibial tuberosity avulsion)	

*POD* postoperative days

**Table 7:** Details of etiology, preoperative and postoperative ROM, type of prosthesis, and complications in the study

Patients	Sex	Age	Side	Etiology	Preoperative ROM (range)	Stiffness	Prosthesis	Postoperative ROM (range)	ROM gain	Complications
1	F	39	L	OA	0 (0)	Complete ankylosis	PS	110 (0–110)	110	
2	M	66	R	OA	0 (0)	Complete ankylosis	PS	130 (0–130)	130	
3	M	45	L	TB	0 (0)	Arthrodesed	VVC	55 (10–65)	55	Pyogenic infection, extension lag
4	F	39	R	Trauma	0 (0)	Complete ankylosis	VVC	70 (0–70)	70	
5	F	58	R	TB	0 (45–45)	Complete ankylosis	PS	110 (0–115)	110	
6	F	62	R	Trauma	0 (10–10)	Arthrodesed	VVC	65 (15–80)	65	Aseptic loosening with polyethylene wear, distal femoral fracture extension lag
7	F	44	L	RA	0 (40–40)	Complete ankylosis	VVC	85 (0–85)	85	
8	M	32	L	TB	0 (15–15)	Arthrodesed	VVC	130 (0–130)	130	Polyethylene dislocation
9	M	66	L	Trauma	0 (50–50)	Complete ankylosis	VVC	15 (15–30)	15	Pyogenic infection, extension lag
10	F	63	R	RA	0 (0)	Complete ankylosis	VVC	70 (15–85)	70	Extension lag
11	F	70	R	OA	0 (0)	Arthrodesed	VVC	125 (0–125)	125	
12	F	83	L	OA	0 (0)	Arthrodesed	VVC	40 (0–40)	40	
13	F	66	L	RA	0 (5–5)	Arthrodesed	VVC	60 (50–110)	60	Patellar fracture with severe extension lag
14	M	74	R	TB	0 (10–10)	Complete ankylosis	VVC	70 (0–70)	70	
15	F	61	L	Pyogenic infection	0 (0)	Arthrodesed	VVC	30 (0–30)	30	

*F* female, *M* male, *OA* osteoarthritis, *TB* tuberculosis, *RA* rheumatoid arthritis, *ROM* range of motion, *PS* posterior-stabilized, *VVC* valgus-varus constrained

## Discussion

The indication for TKA in painless, stiff knees is controversial. The ambulation capacity is not excessively decreased in most cases and the patients may be satisfied with their painless, stable knee. In our cases, all knees were painless. Thirteen patients (86.6%) were able to ambulate without the aid of devices, and two patients (13.3%) used aid devices for ambulation (one a cane, and one crutches).

However, stiff knees lead to a functional deficit and psychological impairment of the patients. The expectations of patients include “better stair-climbing,” “able to move long distance using an automobile or plane,” “easy to sit on a chair”, and “cosmetic reasons”. Patients with a completely ankylosed or arthrodesed knee wanted an operation to obtain an improved life quality. In these knees, TKA can be a treatment option. After the operation, all patients were satisfied with their mobile knee. Even the patients with a marked extension lag stated that they had better functional capacity.

Because soft-tissue adhesion and osteoporosis are frequent in stiff knees, the exposure during surgery is difficult and complications, such as a fracture or an extensor-mechanism injury, are not uncommon. To prevent these complications, an additional procedure may be added to the usual arthrotomy. Ahmed et al. reported good results of TKA using a tibial tubercle osteotomy in patients with rheumatoid arthritis [11]. Twenty-three knees with a flexion contracture of ≥ 15º and/or < 75º range of flexion were treated and showed a good result. In our study group, all knees were exposed using the quadriceps turndown or tibial tuberosity osteotomy. A major concern when using the quadriceps turndown method is the extension lag, and the lengthening procedure may aggravate the extensor weakness. In our study, eight knees showed an immediate postoperative extension lag. It was improved in three knees during a 6-week to 3- month period and five other knees had a residual extension lag at the last follow-up. Four knees showed mild extension lag (10–15º), and one knee showed 50º of lag.

The result of TKA in stiff knees is not equal with that in non-stiff knees. TKA in stiff knees is technically demanding; however, the results are poorer than for non-stiff knees with higher complication rates [12, 13]. Kim et al. reported the results of 86 TKAs in stiff knees with a mean preoperative ROM of 40 (range; 10–50º) [7]. The Hospital for Special Surgery Knee score, KSS, and FS scores improved from a preoperative 42, 11, and 42 points, to a postoperative 84, 90, and 84 points, respectively. The complication rate was 14% (12 knees). In another report, Kim et al. reported TKA conversion for 10 ankylosis knees using a rectus-snip technique [12]. The mean ROM was improved from 9.5º (range; 0–30º)to 78.5 (range; 15–115º). KSS and FS scores were improved from 42.6 (range; 25–70) and 39 (range; 0–60) to 68.6 (range; 41–97) and 66 (range; 40–90), respectively. Only one patient had no improvement in ROM. The complications were: one case of skin-edge necrosis and two cases of radiolucent lines around the tibial component. Rajgopal et al. also reported the results of 115 stiff knees with a ROM between 0 and 20º [9]. Complications were noted in 24 knees (20.9%). Nine knees of extension lag (10–20º), others showed stiffness, flexion contracture, infection, skin necrosis, component loosening, peroneal nerve palsy, and heterotrophic ossification. Naranja et al. reported the results of TKA conversion of 37 previously ankylosed or arthrodesed knees [8]. The average postoperative ROM showed 7º of extension lag and 62º of flexion. The total complication rate was 35% and only 10 patients (29%) were satisfied with no pain and unlimited ambulation. They warned that surgeons should reconsider the risks and benefits of this difficult procedure. Other authors also reported an improved functional result after TKA with the advice to be cautious of the relatively high complication rate [13–16]. In our series, the functional scores (KSS and FS) and ROM improved significantly. The total complication rate was 33.3% (5/15 knees). Four knees underwent revision surgery due to infection (two knees), polyethylene dislocation, and aseptic loosening. In Table 8, our study is compared to other similar studies regarding mean follow-up, ROM gain, functional outcome, and complications. All studies showed satisfaction with the result with improved ROM and functional scores. However, the rates of complications were relatively high in all studies.

**Table 8:** Comparison of results with other studies

	Knees	Mean follow-up period (years)	Mean ROM gain (preoperative to postoperative mean ROM change)	Mean functional score change	Rate and details of complications
Kim et al. (2017) [12]	10	10.2	69 (9.5 to 78.5)	KSS; 42.6 to 68.6 FS; 39 to 66	30% (3 knees) Radiolucent lines around the tibial component (2) and skin edge necrosis (1)
Bahn et al. (2006) [1]	26	6.5	74 (0 to 74)	KSS; 47 to 75	24% (NA) Major complications (4 knees); fracture (1), hematoma needing reoperation (1), peroneal nerve palsy (1), and persistent stiffness (1)
Naranja et al. (1996) [8]	37	7.5	62 (0 to 62)	NA	35% (16 knees) Patellar tendon or tibial tubercle avulsion (3), persistent pain requiring arthrodesis (1), joint stiffness requiring revision or arthrotomy for lysis of scar (2), aseptic loosening requiring revision (5), and deep infection (5)
Our current study	15	15.4	77.6 (0 to 77.6)	KSS; 36 to 76.9 FS; 50.9 to 67.2	33% (5 knees) Periprosthetic joint infection (2), Polyethylene-insert dislocation (1), severe extension lag after patella fracture (1), and aseptic loosening (1)

*ROM* range of motion, *KSS* Knee Society Knee score, *FS* Knee Society Function score, *NA* not available

There are some limitations in our study. The study design is retrospective, we used different types of prostheses, and the number of included patients was small.

## Conclusions

The results of TKA for painless, completely ankylosed or arthrodesed knees were satisfactory with improved ROM and quality of life. Although some patients had mild pain postoperatively, they were satisfied with the results. However, our study recommends that surgeons should consider the high rate of complications.

## Data Availability

Not applicable.
